# Cognitive and Personality Components Underlying Spoken Idiom Comprehension in Context. An Exploratory Study

**DOI:** 10.3389/fpsyg.2018.00659

**Published:** 2018-05-01

**Authors:** Cristina Cacciari, Paola Corrardini, Fabio Ferlazzo

**Affiliations:** ^1^Department of Biomedical, Metabolic and Neural Sciences, University of Modena-Reggio Emilia, Modena, Italy; ^2^Center for Neuroscience and Neurotechnology, University of Modena-Reggio Emilia, Modena, Italy; ^3^Department of Psychology, University of Rome-Sapienza, Rome, Italy

**Keywords:** idiom comprehension, individual differences, working memory, inhibitory control, vocabulary, cognitive flexibility, personality, predictability

## Abstract

In this exploratory study, we investigated whether and to what extent individual differences in cognitive and personality variables are associated with spoken idiom comprehension in context. Language unimpaired participants were enrolled in a cross-modal lexical decision study in which semantically ambiguous Italian idioms (i.e., strings with both a literal and an idiomatic interpretation as, for instance, *break the ice*), predictable or unpredictable before the string offset, were embedded in idiom-biasing contexts. To explore the contributions of different cognitive and personality components, participants also completed a series of tests respectively assessing general speed, inhibitory control, short-term and working memory, cognitive flexibility, crystallized and fluid intelligence, and personality. Stepwise regression analyses revealed that online idiom comprehension was associated with the participants' working memory, inhibitory control and crystallized verbal intelligence, an association modulated by idiom type. Also personality-related variables (State Anxiety and Openness to Experience) were associated with idiom comprehension, although in marginally significant ways. These results contribute to the renewed interest on how individual variability modulates language comprehension, and for the first time document contributions of individual variability on lexicalized, high frequency multi-word expressions as idioms adding new knowledge to the existing evidence on metaphor and sarcasm.

## Introduction

Idioms still represent a challenge to theories of language processing after more than 40 years from the first psycholinguistic study on idiom comprehension (Bobrow and Bell, 1973). Idioms belong to the broad class of over-learned sequences of words (multi-word expressions, MWEs, for overviews see Siyanova-Chanturia, 2013; Cacciari, 2014, among others) whose representations are stored in semantic memory. In the last decade, idiomatic expressions have gained the attention of several researchers out of the community of figurative language scholars mostly because what was initially seen as an oddness—the fact that idioms convey a figurative interpretation (and some of them a literal meaning as well) not fully determined by a compositional syntactic and semantic analysis of their component words—is now considered an interesting aspect that must be explained by language processing models. In addition, idioms represent a good test case for the role of distributional properties of language in comprehension since idiom composing words are bound together with a probability much higher than they would have in freely formed sentences (Vespignani et al., 2010). Thus, studying the processing mechanisms underlying MWEs may provide relevant insights about how the brain stores and processes lexico-semantic knowledge and how semantic storage and processing interact with the frequency with which words co-occur in the linguistic environment (Molinaro and Carreiras, 2010; Vespignani et al., 2010).

Several models have been proposed to account for idiom processing (see below). Notwithstanding, the complex cognitive architecture that stands behind the relatively easy and fast comprehension of familiar idioms has not yet been singled out in full details. Can we take for granted the assumption that the cognitive architecture underlying language comprehension is precisely the same no matter whether the input is literal or idiomatic? The answer is that we do not know yet. There are reasons to suspect a different involvement and/or weight of some cognitive components (e.g., working memory or executive function) when the input is an idiomatic string compared to when it is a matched literal string. In fact, idioms are somewhat “special” in that they behave at the same time as partially compositional string of words (as any other piece of language) and as non-compositional string of words: while the meaning of a sentence is obtained by merging individual word meanings as the message unfolds, in idiomatic sentences what must be merged comprises unitary sequences of co-occurring words (Cacciari and Corradini, 2015). In addition, when idioms also have a well-formed literal meaning (e.g., *break the ice*), the reader/listener has to decide between competing interpretations favoring the one contextually relevant and inhibiting already processed and actually irrelevant information associated to individual word meanings.

Interestingly, the idiom processing time advantage found with a variety of behavioral tasks (e.g., Swinney and Cutler, 1979; Cacciari et al., 2007; Vespignani et al., 2010; Siyanova-Chanturia et al., 2011; Canal et al., 2017) is accompanied by brain-imaging evidence suggesting a more complex picture. These studies consistently reported a stronger and more widespread activation of the language network for idioms, in particular in the Inferior Frontal and Middle Temporal Gyri (e.g., Zempleni et al., 2007; Mashal et al., 2008; Romero Lauro et al., 2008; Boulenger et al., 2009, 2012) rather inconsistent with the idea that the comprehension of ready-to-go idiomatic meanings is relatively undemanding to the processing system^1^. In sum, idiom processing seems to be at the same time fast but more resource-demanding than the processing of comparable literal sentences. This increased workload on the language system may reflect the need to process at least part of the literal meanings of the idiom constituent words, retrieve the idiomatic meaning from long-term semantic memory, select between potentially competing interpretations, and integrate the idiom meaning in context while suppressing idiom-irrelevant word meanings. Whether these mechanisms operate sequentially or at least partially in parallel depends on several factors including the idiom type, familiarity, predictability before offset and prior contextual information.

Here, we adopted an individual difference perspective to explore the contributions of components underlying the cognitive architecture necessary to process idiomatic expressions in adults, namely general speed, inhibitory control, working memory, cognitive flexibility, vocabulary knowledge, fluid intelligence, and personality. Specifically, using a correlational approach, we explored whether and how individual differences in these components are associated with the moment-by-moment context-driven comprehension of spoken idioms. To the best of our knowledge, the contribution of personality factors to idiom comprehension in healthy participants has been largely ignored. Therefore, we used two tests assessing, respectively, current symptoms of anxiety and propensity to be anxious (STAI-Y) and components of the personality structure (Big Five Observer). Measuring state and trait anxiety may reveal whether or not increased emotional arousal associated to the specific event (the experiment) or to the individual's personality may lead participants to recruit more attentional and processing resources speeding up mental processing (as documented, in general, by Svenson and Maule, 1993; Breznitz and Berman, 2003). As to the Big Five Observer, recently it has been documented an association of some of the components identified by this test with creativity and metaphor comprehension (for an overview, see Batey and Furnham, 2006). It could be interesting to explore whether associations of this type could generalize to conventionalized forms of figurative language as idioms.

## Models of idiom processing

Decades ago, Bobrow and Bell (1973) proposed that idioms are semantically empty long words accessed as such from the mental lexicon (Bobrow and Bell, 1973; Swinney and Cutler, 1979; Gibbs, 1980). Consistently, the *Lexical Representation hypothesis* (LRH; Swinney and Cutler, 1979) proposed that idiom meaning is directly retrieved from semantic memory and not elaborated via linguistic processing. The computation of the literal and idiomatic meaning is simultaneously initiated upon occurrence of the first idiom component word. Since retrieving the idiomatic meaning takes less time than computing the compositional meaning of the string, the idiom meaning becomes available before the literal meaning. In more recent years, evidence against this “Lexical-look up” approach has accumulated (e.g., Cacciari and Tabossi, 1988; Titone and Connine, 1994; Peterson et al., 2001; Tabossi et al., 2005; Sprenger et al., 2006; Cacciari et al., 2007; Libben and Titone, 2008; Fanari et al., 2010; Vespignani et al., 2010; Holsinger and Kaiser, 2013; Canal et al., 2017) leading to “Non-Lexical Hybrid” models. The *Configuration Hypothesis* (CH, Cacciari and Tabossi, 1988; Tabossi and Zardon, 1993; Vespignani et al., 2010; Cacciari and Corradini, 2015) provided an influential model of how spoken idiom comprehension unfolds in time. According to the CH, idioms are incrementally processed word by word, just like any other linguistic string, until enough information has accumulated to signal the presence of an idiom. Only at this point the idiomatic meaning is retrieved from semantic memory. Hence, the point at which the string is recognized as a known idiom determines how early the idiomatic meaning is activated. This recognition point is established in *probabilistic terms* as the point after which the probability of a fragment to be continued idiomatically is extremely high. Idioms can therefore be predictable or unpredictable before the string offset depending on how many idiom word constituents must be processed before the presence of an idiom is recognized or highly expected (Vespignani et al., 2010). Idiom activation can be influenced by the information conveyed by previous context (Peterson et al., 2001; Fanari et al., 2010) since even a minimal idiom-biasing context can anticipate the idiom recognition point. In this case, the idiomatic meaning may be already activated at the end of the idiom string regardless of idiom predictability.

## The role of individual differences in figurative language processing

As recently pointed out by Kidd et al. (2018), despite a long-standing assumption downplaying the role of individual differences in cognition, it is becoming increasingly clear that there are important variations among speakers in language acquisition and processing at any age and across the lifespan. These may stem from different sources since language comprehension involves many and diverse perceptual skills and cognitive processes that interact among them and with language experience (Farmer et al., 2017). The renewed interest on how individual differences modulate spoken and written language comprehension goes beyond what was traditionally observed in language-impaired populations (e.g., Gernsbacher and Faust, 1991; Just and Carpenter, 1992; Miyake et al., 1994; Hannon and Daneman, 2001; Verguts and De Boeck, 2002; McNamara and McDaniel, 2004; Andrews et al., 2017 among others; for overviews see Farmer et al., 2012; Andrews, 2015; Kidd et al., 2018).

Surprisingly enough, this new wave of studies on individual variability has predominantly concerned literal language almost ignoring figurative language, despite its pervasiveness. The few studies on individual differences in figurative language comprehension have predominantly concerned the comprehension and/or production of metaphorical sentences in adults (e.g., Kazmerski et al., 2003; Chiappe and Chiappe, 2007; Pierce et al., 2010) and/or children and adolescents (e.g., Nippold and Martin, 1989; Johnson, 1991; Nippold et al., 2001; Qualls and Harris, 2003; Qualls et al., 2003; Carriedo et al., 2015). Metaphor comprehension requires a high level of intellectual ability, processing capacity and inhibitory control (e.g., Kintsch, 2000, 2001; Kazmerski et al., 2003; Carriedo et al., 2015). In fact, all other things being equal, comprehending a metaphor implies the abstraction of common features between target and source domains, the blend of often distant semantic domains, and the capacity to selectively identify, within the source domain, what is relevant to the target and suppress irrelevant properties (e.g., that sharks have fins for “That lawyer is a shark”). This inhibitory control requires a high WM capacity for maintaining, momentarily, two interpretations for a same utterance (the literal and the metaphoric), deciding which aspects of the literal meaning are metaphor-relevant (e.g., that sharks are voracious), and updating the ongoing sentential representation (Chiappe and Chiappe, 2007; Carriedo et al., 2015; Olkoniemi et al., 2016).

Consistently, some studies reported that less-skilled comprehenders less efficiently suppress interfering and/or irrelevant information, no matter whether they are adults (Gernsbacher and Faust, 1991) or children (De Beni et al., 1998; Cain and Oakhill, 2006). In a study on metaphors with participants aged from 11 to 21 years, Carriedo et al. (2015) found that the importance of executive function, a fundamental part of the cognitive architecture underlying suppression of interfering information, is evident only in adolescence. Chiappe and Chiappe (2007) found that WM capacity, inhibitory control, and vocabulary knowledge contribute to the speed and quality of the interpretation assigned to metaphorical statements in adults. Moreover, participants scoring higher in WM measures also produced more apt metaphors than participants scoring lower. According to the authors, two types of factors are associated with the ability to comprehend and produce metaphors: information processing factors related to the executive mechanisms of WM, and experiential factors related to crystallized verbal knowledge. Consistently, Pierce et al. (2010) showed that high WM skills are associated with more accurate and faster recognition of the non-literal meaning of metaphorical statements.

In an eye-tracking study, Columbus et al. (2016) tested whether familiarity and context modulate the comprehension of metaphorically and literally intended verbs as a function of individual differences in executive control (the same participants also performed an idiom comprehension experiment, see below). Familiarity of metaphorical verbs affected how much time participants spent reading the verb on first pass regardless of prior context. Interestingly, readers with high executive control spent more time fixating the verbs presumably to integrate them in the unfolding sentential interpretation. In contrast, individuals with low executive control did not spend a similar extra time but had longer reading times later on in the metaphor region. This suggests that executive control skills influence the rapidity with which semantic commitment to the verb interpretation is made with effects persisting until the end of the sentence. Another eye-tracking study (Olkoniemi et al., 2016) examined the role of individual differences in WM capacity, need for cognition, use of emotional information, and theory of mind in comprehending sarcasm, metaphor, and literal sentences. Individual differences in WM capacity and need for cognition modulated both the comprehension of metaphoric and sarcastic statements, but efficiency in using emotional information only played a role for sarcasm.

A related line of investigation has tested the roles of fluid intelligence and creativity in comprehending and producing metaphors. Silvia and Beaty (2012) found that measures of fluid intelligence (with tasks mostly testing non-verbal inductive reasoning) were associated with the capacity to produce creative metaphors. Participants with high fluid intelligence produced more creative metaphors than those scoring lower. In another study, Beaty and Silvia (2013) explored the roles of fluid and crystallized intelligence, and retrieval capacity in metaphor production. They observed differential contributions of cognitive skills depending on the conventionality or creativity of the metaphors produced. Specifically, fluid intelligence and retrieval capacity had little association with the production of conventional metaphors, and medium-size association with crystallized intelligence. This suggests that the generation of conventional metaphors primarily recruits prior knowledge and minimally executive resources, while the production of creative metaphors involves sophisticated retrieval mechanisms, as suggested by the observed important contribution of fluid intelligence.

We now turn to the even fewer studies investigating individual differences in idiom comprehension. Cacciari et al. (2007) tested the contribution of individual speed of processing in comprehending ambiguous idioms, predictable or unpredictable before offset, using a cross-modal lexical decision paradigm. Fast and slow participants were equally fast for predictable idioms. Participants with slower speed of processing also required more perceptual information before recognizing unpredictable idioms. In contrast, fast participants responded equally quickly to targets associated to the two types of idiom. With a response deadline, all participants activated unpredictable idiom meanings equally quickly. Presumably, the response deadline speeded up mental processing and changed the allocation of time and processing resources (Svenson and Maule, 1993; Breznitz and Berman, 2003). Reading acceleration in fact extends attention span, reduces distractibility, helps to overcome some capacity limitations of short term and working memory, and increases word retrieval from the mental lexicon (Breznitz and Berman, 2003). In the eye-tracking study described above, Columbus et al. (2016) did not find any association of executive control with idiom processing, at variance with neuropsychological evidence showing the centrality of executive function to idiom comprehension.

## Rationale of the study

The main goal of this study was to explore the different contributions of cognitive and personality components to moment-by-moment spoken idiom comprehension when supported by contextual information. To assess idiomatic meaning activation we used a paradigm, the cross-modal lexical decision task, that has been fruitfully used in many previous studies on idiom comprehension in and out of context (e.g., Cacciari and Tabossi, 1988; Titone and Connine, 1994; Tabossi et al., 2005; Cacciari et al., 2007; Fanari et al., 2010). In this paradigm, participants listen to a sentence and judge whether a visually presented target word is or is not a word. In this study, the prime is an idiom-biasing sentence ending with an idiom and the target word either a word semantically associated with the idiomatic meaning or an unrelated, control word (see the Method section for the target selection procedure). Semantic priming occurs whenever there is more efficient processing of the target word when preceded by a related context, in this case the idiom-biasing sentence, which means faster decision times for the idiomatic than for the control target. In this study, we analyzed whether measures of general speed, inhibitory control, short-term and working memory, cognitive flexibility, crystallized and fluid intelligence, and personality are associated with and predicted the cross-modal lexical decision times to predictable and unpredictable ambiguous idioms. Since ambiguous idioms possess both a literal and an idiomatic interpretation, the idioms tested in this study were preceded by idiom-biasing contexts that clarified which one of the two interpretations was contextually appropriate. In line with the CH, we assume that any cues to a non-literal sentential interpretation are likely to anticipate the point at which a fragment can bring to mind an idiom, triggering idiom meaning activation. In fact, as previous studies have shown (e.g., Peterson et al., 2001; Cacciari et al., 2007; Fanari et al., 2010), even a short context preceding an idiom can bias toward the idiomatic interpretation. Given that the idiom strings of this study were embedded in idiom-biasing contexts, we expect participants to activate the idiomatic meaning of both predictable and unpredictable idioms. Nonetheless, the activation of unpredictable idioms may require longer response times than for predictable idioms (Cacciari and Corradini, 2015). In fact, the notion of predictability captures the fact that, upon hearing part of a string, participants may recognize it as belonging to a known idiom. Although idiom-biasing contexts speed up idiom recognition, identifying an unpredictable idiom (whose recognition point out of context is after the idiom string offset) may require the processing of more input than identifying a predictable idioms. Differently, listening to a smaller part of the string may suffice to actively pre-activate the idiomatic meaning of predictable idioms. In this study, responding to idiomatic targets of predictable idioms should be the easiest and fastest (given the joint effects of idiom familiarity, contextual bias, and predictability), followed by the idiomatic targets of unpredictable idioms, then by the control targets of predictable and unpredictable idioms that are both idiom-unrelated and not primed by idiom-biasing contexts.

## Experiment and testing

### Method

#### Participants

Sixty-four students volunteered to participate in the study (23 male and 41 female; mean age = 21.9 years, *SD* = 3.4). All were Italian native speakers with normal or corrected-to-normal vision, unaware of the aim of the study. This study was carried out in accordance with the recommendations of the “Italian Association of Psychology” (AIP) Ethical Guidelines (*Codice Etico*: www.aipass.org/node/11560) and with the standard procedures of the University of Modena-Reggio Emilia. All subjects gave written informed consent in accordance with the Declaration of Helsinki.

#### Materials

##### Norming phase for the cross-modal lexical decision experiment

*Idiom familiarity and knowledge of the idiomatic meaning*. Eighty idioms with both an idiomatic and a literal interpretation (ambiguous idioms) were selected from a list of Italian idioms. Twenty-five university students were asked to rate the familiarity of each idiom on a seven-point scale (from *1*: never heard to *7*: heard very often) reported under each idiom. At the end of the questionnaire, they went back to the beginning and paraphrase each idiom. Since the task was rather demanding in time, the idioms were split into two questionnaires. We selected 48 idioms highly familiar (*M* = 5.75, *SD* = 0.54) and correctly paraphrased by 95% of the participants.

*Age of acquisition (AoA)*. Since the AoA of idioms correlates with idiom familiarity and knowledge of the idiomatic meaning (Tabossi et al., 2011; Bonin et al., 2013), and is a reliable predictor of paraphrase verification times (Bonin et al., 2013), we selected predictable(P) and unpredictable (UP) idioms with similar AoAs. The AoA was evaluated using a questionnaire listing the 48 idioms with a 7-point rating scale under each idiom (1: 0–2 years, 2: 3–4 years, 3: 5–6 years, 4: 7–8 years, 5: 9–10 years, 6: 11–12 years, 7: more than 13 years). Twenty-five students were asked to estimate the age at which they had learned each idiom. Participants reported that they learned the idioms, on average, between 10 and 11 years (*M* = 5.28; *SD* = 1.2), in line with what has been shown in the idiom acquisition literature (Levorato and Cacciari, 1999).

*Idiom recognition point*. In order to assess the point at which the idiomatic nature of each string was identified, a questionnaire was prepared with 48 minimal contexts containing the idioms without the last constituent (e.g., *the boy broke the …*) and 48 filler fragments of similar length and structure. Twenty-five students were asked to complete each sentence with the first word that came to mind. The idioms that were completed idiomatically with a cloze probability of at least 70% were considered as predictable (P idioms) and those that received <30% of idiomatic completions as unpredictable (UP idioms). To ensure that the completions were not due to literal interpretations, each participant was also asked to provide a meaning paraphrase of each sentence. Participants always provided idiomatic paraphrases. Twenty-eight idioms were selected, 14 predictable and 14 unpredictable, balanced for familiarity and AoA (see Tables 1–3).

**Table 1:** Mean values, standard deviations (in parentheses), and statistics of the norming of the psycholinguistic variables of predictable and unpredictable idioms.

	**Predictable idioms**	**Unpredictable idioms**	**Statistics**
Idiom recognition	84.7% (8.31%)	10.7% (8.45%)	*t*_(26)_ = 10.055, *p* = 0.0001
Familiarity	6.02 (0.57)	5.86 (0.67)	*t*_(26)_ = 0.695, *p =* 0.493
AoA	4.96 (0.47)	5.09 (0.69)	*t*_(26)_ = 0.567, *p =* 0.575
Meaning dominance	3.73 (0.53)	3.69 (0.50)	*t*_(26)_ = 0.178, *p =* 0.86
Idiomatic target association	4.13 (0.46)	4.0 (0.64)	*t*_(26)_ = 0.608, *p =* 0.548
Idiomatic target length	9.07 (2.2)	8.07 (2.33)	*t*_(26)_ = 0.413, *p =* 0.663
Idiomatic target syllables	3.79 (1.12)	3.5 (0.76)	*t*_(26)_ = 0.789, *p =* 0.473
Idiomatic target AoA	3.89 (0.84)	3.5 (0.72)	*t*_(26)_ = 1.137, *p =* 0.266
Idiomatic target written frequency (fpmw)^*^	46.94 (62.7)	28.83 (48.21)	*t*_(26)_ = 0.857, *p =* 0.399
Idiomatic target familiarity	4.88 (0.53)	4.73 (0.57)	*t*_(26)_ = 0.685, *p =* 0.499
Control target length	8.57 (2.03)	8.21 (2.58)	*t*_(26)_ = 0.408, *p =* 0.687
Control target syllables	3.64 (0.93)	3.5 (1.09)	*t*_(26)_ = 0.274, *p =* 0.712
Control target AoA	3.77 (1.07)	4.12 (1.05)	*t*_(26)_ = 0.869, *p =* 0.393
Control target written frequency (fpmw)^*^	60.76 (62.6)	26.96 (24.45)	*t*_(26)_ = 1.93, *p =* 0.07
Control target familiarity	4.94 (0.75)	4.46 (1.15)	*t*_(26)_ = 1.297, *p =* 0.206
Lex. Dec. Times Idiomatic target	731 ms (80)	777 ms (94)	*t*_(26)_ = 1.388, *p =* 0.177
Lex. Dec. Times Control target	726 ms (54)	776 ms (88)	*t*_(26)_ = 1.769, *p =* 0.089
Number of words of Contexts	14.57 (0.94)	15.21 (1.05)	*t*_(26)_ = 1.708, *p =* 0.10

* *Written frequency was calculated on ITAWaC (Baroni et al., 2009) and expressed as frequency per million of words (fpmw)*.

**Table 2:** Mean values, standard deviations (in parentheses), and statistics of the norming of the psycholinguistic variables of Idiomatic and Control targets.

	**Idiomatic targets**	**Control targets**	**Statistics**
Number of characters	8.89 (2.25)	8.39 (2.28)	*t*_(54)_ = 0.825, *p* = 0.413
Number of syllables	3.64 (0.95)	3.57 (1.0)	*t*_(54)_ = 0.274, *p* = 0.785
AoA	3.72 (0.78)	3.95 (1.06)	*t*_(54)_ = 0.917, *p* = 0.363
Written frequency (fpmw)^*^	30.88 (55.64)	43.85 (48.6)	*t*_(54)_ = 0.428, *p* = 0.67
Familiarity	4.81 (0.55)	4.7 (0.99)	*t*_(54)_ = 0.485, *p* = 0.629

* *Written frequency was calculated on ITAWaC (Baroni et al., 2009) and expressed as frequency per million of words (fpmw)*.

**Table 3:** Examples of experimental materials in Italian and with word-by-word English translations (in parentheses).

**PREDICTABLE IDIOMS**
Il cliente ha ascoltato l'offerta del venditore e ha deciso di prenderla al volo. (The customer listened to the seller's offer and decided to take it on the fly).
Idiomatic target: OCCASIONE (CHANCE)
Control target: DESTINO (DESTINY)
Quando Luisa si è accorta che aveva perso l'antico anello si è strappata i capelli. (When Luisa realized that she had lost the old ring, she torned her hair).
Idiomatic target: DISPERAZIONE (DESPAIR)
Control target: RELAZIONE (RELATIONSHIP)
**UNPREDICTABLE IDIOMS**
Lucia ha fatto il concorso nella sua università perché sapeva di giocare in casa. (Lucia did the competition at her university because she knew she was playing at home).
Idiomatic target: VANTAGGIO (ADVANTAGE)
Control target: SVOLTA (TURN)
Sara stava raccontano una favola alla nipotina ma si accorse di aver perso il filo. (Sara was telling a story to her granddaughter but realized she had lost the thread).
Idiomatic target: DISTRAZIONE (DISTRACTION)
Control target: NAZIONE (NATION)

*Meaning dominance*. To test whether the dominant meaning of the idiom strings was literal or figurative, 25 participants were asked to rate the extent to which each idiom string was heard/used in a literal or figurative sense (1: mostly literally to 5: mostly figuratively). The figurative meaning was similarly perceived as the dominant one in P and UP idioms.

*Target selection*. To select the idiomatic visual targets, 15 participants were asked to provide a word semantically associated to the idiomatic meaning of the string. The words more frequently associated to each idiom were listed under the idiom string in a written questionnaire. Fifteen students were asked to rate on a 5-point scale the extent to which each word was associated to the figurative meaning of the idiom string (from 1 indicating not at all associated, to 5 strongly associated). The words with the highest association rating were used as visual targets. The mean association ratings for the targets of P and UP idioms were similar. To control that the selected targets were not associated with the last word of the string, 10 students were presented with the last word of each string and were asked to produce the first associated word that came to mind. None of the experimental targets was generated. The targets for P and UP idioms were balanced for number of characters and syllables, AoA, written frequency, and familiarity (see Table 3 for examples, and the Appendix for the idioms and targets list).

All control targets had an abstract meaning as the idiomatic targets, were not produced in the selection of idiomatic targets nor were they associated to idioms. Idiomatic and control targets were balanced for number of characters and syllables, AoA, written frequency, and familiarity. The control words associated to P and UP idioms were balanced for number of characters and syllables, familiarity, AoA, and written frequency.

A control experiment was run to determine whether the lexical decision times for the targets of P and UP idioms were comparable when presented in isolation. The 28 target words were included in a list of 112 items (56 words, i.e., 28 idiomatic and 28 control targets, and 56 legal non-words). The filler words were all abstract. Each stimulus was visually presented in a randomized order to 15 participants who performed a visual lexical decision task. Lexical decision times were statistically similar for idiomatic and control targets; for idiomatic targets associated to P and UP; and for controls associated to P and UP idioms.

*Sentential contexts*. The 28 idiom strings were inserted in contexts orienting the interpretation toward the figurative sense of the string. The average number of words composing them was 14.89 (*SD* = 1.03). The idiomatic contexts associated to P and UP idioms had a similar number of words.

In addition to the 28 experimental sentences, 84 filler sentences of the same length (*M* = 14.94, *SD* = 1.22) and similar syntactic structures, but without any figurative expressions, were created. A visual target was paired to each filler sentence: 56 targets were legal non-words and 28 were words, so that we had an equal number of words and non-words within each list. The filler target words had on average the same number of syllables of the experimental words. Fourteen of the filler words were semantically associated to the sentence in order to avoid that participants developed a response set to the experimental trials. All filler words had an abstract meaning.

Two lists were created so that each idiom was presented only once, either associated to the idiomatic target or with the control target. Participants were randomly associated to one of the two lists. Each list contained 112 sentences in a quasi-randomized order: 28 experimental sentences (14 idiomatic sentences followed by idiomatic targets, 14 idiomatic sentences followed by control targets) and, interspersed among the experimental sentences, the 84 filler sentences. The experiment was preceded by a practice session formed by 10 different literal sentences, followed by a word in half of the cases and by a legal non-word in the other half, that were not further analyzed.

The stimuli were presented on an IBM-compatible personal computer using DMDX software (Forster and Forster, 2003). A male speaker digitally recorded the sentences. A cue, inaudible to the participants, was placed in the sound files to specify the position of the target and caused the program to display it on the screen. In all the sentences the target appeared at the offset of the sentence-final word.

A recognition test on the sentences heard during the experiment was prepared to verify that participants had actually paid attention to the sentences. The test was administered immediately after the experimental session and was composed by 28 sentences printed on separate cards. Fourteen of these sentences had appeared in the experiment as filler sentences and 14 had not, but were similar in length and structure to the filler sentences. Participants were given the 28 cards and were asked to pick out those presented during the experiment. A threshold of 75% of correct recognitions was set for inclusion of a participant's data in the statistical analyses.

##### Testing of individual differences

The cognitive and personality components tested in this study were assessed with the following tasks:

*General speed*: Simple Reaction Time task. Participants were asked to press as fast and accurately as possible a specific response button (CTRL left or right depending on the dominant hand) each time a star appeared on the screen. The randomly distributed inter-trail intervals were 600, 900, 1,200, and 1,500 ms. There was a practice session of 10 trials followed by 80 experimental trials. We recorded the response times between the onset of the stimulus and the response. Response times exceeding 1,500 ms were considered as errors.

*Inhibitory control*: (1) Go-No Go task. Participants were asked to press the space bar as fast and accurately as possible when a triangle (Go stimulus) appeared on the screen and refrain from responding when a circle appeared (No-Go stimulus). There were 200 trials, of which 160 were Go trials and 40 No-Go trials, with randomly distributed inter-trial intervals of 600, 900, 1,200, and 1,500 ms. We recorded the response times to GO trails, omissions (i.e., no response to GO trials) and intrusions (incorrect response to NO-GO trials); (2) Stroop task. Participants were presented with letter strings for which they had to name the color. Each letter string could belong to either one of three conditions: congruent (e.g., VERDE, written in a matching hue, green), incongruent (e.g., VERDE written in a mismatching hue, red), or neutral. This condition was represented by the letter string XXXX written in red, blue or green. Congruent and incongruent color words were VERDE, BLU, and ROSSO. There were 96 trials, with 24 congruent trials, 24 incongruent trails, and 48 neutral trials (16 for each color). We recorded the latency between presentation of the item and onset of the participant's response. The inter-trial interval was 2,000 ms. Prior to the experimental trials, there were 12 trails of practice in which participants named the color white in which VERDE, BLU, or ROSSO were written on the screen. For each participant, we computed a facilitation effect (the mean RT to congruent trails minus the mean RT to neutral trials), an inhibition effect (neutral RT minus incongruent RT), and a maximal inhibition effect (incongruent RT minus congruent RT), (3) Intrusions errors in the Listening Span test (see below). They provide an estimate of inhibitory control since participants are required to use it to reduce the accessibility of non-final words, prevent their recall and therefore produce fewer intrusions (De Beni and Palladino, 2000).

*Short-Term and Working Memory*: (1) Forward Digit Span Test and Backward Digit Span Test (from the *Wechsler Adult Intelligence Scale Revised*, Wechsler, 1981; Italian Version: Laicardi and Orsini, 1998). These tests assess the serial recall of digit strings. The scores are based on the higher number of digits recalled in the correct forward and backward orders. Each test starts with a string composed by three digits and continues by adding one digit until the participant fails to recall the string twice; (2) Backward counting: Participants were asked to count backward from 100 to 1 as fast and accurately as possible. We recorded the total latency; (3) Listening Span (Daneman and Carpenter, 1980; Italian Version: Pazzaglia et al., 2000). This test requires the memorization of the last item of each sentence (auditorily presented) within sequences of sentences (composed by between 6 and 12 words) with 20 sequences including 2, 3, 4, 5, and 6 simple sentences (four sequences in each case). Half of the sentences are “true” and half “false.” Participants were asked to listen to each sentence and answer whether it was “true” or “false.” At the end of the sequence, participants were asked to recall the last word of each sentence. We considered three indices: the *listening span*, that is the highest level at which all the final words were recalled in the correct order; the *number of recalled words*, that is the total number of items correctly recalled in the correct order; and the *number of intrusions*, that is the number of non-final words that were incorrectly recalled but that belonged to the set of presented sentences. It has been proposed (for overviews, see Farmer et al., 2012, 2017) that reading/listening span tests provide measures of language processing skills driven by linguistic experience rather than only of verbal WM.

*Cognitive Flexibility and Crystallized Verbal Intelligence*: (1) Phonemic and Semantic Fluency Tests (Italian Version: Novelli et al., 1986). In the Phonemic fluency test, participants were asked to produce as many words beginning with given letters (in Italian, F, P, L) as possible in a time interval (60′′ for each letter). In the Semantic fluency test, individuals produce as many members of given stimulus categories (car brands, fruits, and animals) as possible in a time interval (60′′ for each category). The final scores of Semantic and Phonetic fluency are obtained by summing up the number of items produced within the time interval for each letter or category. According to Troyer et al. (1997), the fluency performance is based on two distinct cognitive operations: Clustering (i.e., the production of words within semantic and phonetic categories) and switching (i.e., the ability to shift between clusters) both assessing cognitive flexibility. This test is considered to provide an estimate of executive function (Troyer et al., 1997; Lezak et al., 2004); (2) Vocabulary knowledge: Vocabulary subtest of the *WAIS-R*. Participants were asked to define the meaning of 40 items. Correct definitions were assigned 2 points, partially correct 1 point, and no response and incorrect definitions 0 points. The Vocabulary subtest is thought to estimate the global verbal intelligence function related to already acquired knowledge (namely, crystallized intelligence; Lezak et al., 2004). This test also provides a possible estimate of lexical access skill in adults (Kidd et al., 2018).

*Fluid Intelligence*: Raven Progressive Matrices (RPM, Raven, 1960). RPM is a non-verbal test that provides an estimate of non-verbal fluid intelligence by testing spatial, abstract reasoning. Participants were presented with a booklet comprising four series of visual geometric forms (of 12 items each, of increasing complexity) with a missing piece, and were given six to eight choices to pick from and fill in the missing piece (within a total time limit of 30 min). The performance is measured in terms of total number of responses and number of correct responses for the matrices.

*Personality*: (1) Spielberger State-Trait Anxiety Inventory (STAI-Y, Spielberger et al., 1970). It is used to measure, via self-report, the presence and severity of current symptoms of anxiety and a generalized propensity to be anxious. The first subscale, the State Anxiety Scale (formed by 20 items), evaluates the current state of anxiety, asking how respondents feel “right now,” using items that measure subjective feelings of apprehension, tension, nervousness, worry, and activation/arousal. The second subscale, the Trait Anxiety Scale (formed by 20 items), measures relatively stable aspects of anxiety proneness, including general states of calmness, confidence, and security. Each item of the two subscales is rated on a 4-point scale. Higher scores indicate greater anxiety; (2) Big Five Observer (BFO, Italian Version: Caprara et al., 1993). This personality test is inspired by the BIG FIVE model of personality (Costa and McCrae, 1992) that has identified five dimensions of personality: 1. Extroversion (i.e., interest in people and events, ability to enjoy, assertiveness, activeness, a desire to communicate, and emotional feelings); 2. Agreeableness (compassion, niceness, gentleness, confidence in others, trust, and warmth); 3. Conscientiousness (high level of organization and perseverance during task-oriented activities, diligence, responsibility, and achievement); 4. Neuroticism (lack of stability, worry, fear, depression, and mood changes); 5. Openness to Experience (creativity, imagination, rapid grasp, curiosity). The test is composed by 40 pairs of opposite adjectives (eight for each of the dimensions). Participants were asked for a self-evaluation on each adjective pair using a seven-point Likert scale.

## Procedure

In the cross-modal lexical decision experiment, participants were tested individually in a sound-attenuated room. They sat at a distance of ~65 cm from the computer screen. The target word appeared in the center of the screen written in black characters (GENEVA 18) on a white background and remained on the screen until participant response. If no response was provided in 4,000 ms, the target disappeared and the next trial appeared. The participant response was followed by an interval of 900 ms before the subsequent sentence began. The experimental instructions were displayed on the screen and then repeated by the experimenter before the training session. Participants were informed that a letter string would appear at the end of each sentence. They had to decide whether it corresponded or not to an Italian word and press an appropriately labeled response key (CTRL key on the right = word and CTRL key on the left = non-word for right-handed participants, the opposite for left-handed ones). After 10 training trials, the experiment began. Participants were instructed to pay attention to the sentences because they would be asked some questions at the end of the session. Immediately after the experiment, participants performed the recognition test. The experiment lasted on average 25 min.

Testing and cross-modal lexical decision experiment were individually performed in two different sessions taking place a week after the other. The order of testing and experiment was quasi-randomized across participants. In one session, participants performed the following tests in this order: Listening Span test, Simple reaction times, Go-No Go task, Digit Span, Semantic and Phonetic fluency, Vocabulary; in another session, they carried out the cross-modal lexical decision experiment, the Stroop test, RPM, STAI-Y, and BFO.

## Data analysis

We conducted analyses of variance (ANOVAs) on the average reaction times of correct responses for condition to the cross-modal lexical decision experiment considering both participants and items as random factors. In the by-participant analysis (indicated by subscript 1), Idiom type (Predictable vs. Unpredictable) and Target (Idiomatic vs. Control) were within-subject factors. In the by-item analysis (indicated by subscript 2), Idiom Type was a between-subject factor and Target a within-subject factor.

To explore whether individual difference in the cognitive and personality components were associated with the comprehension of idioms, we computed: (1) Pearson correlations between the participants scores in each test and their correct average response times to idiomatic and control targets in predictable and unpredictable idioms; to idiomatic and control targets regardless of idiom type; and to predictable and unpredictable idioms regardless of target type (see Table 4). We only report and comment significant bivariate correlations; (2) hierarchical regressions on the average response times to correct answers using blockwise entry. Six predictor variables (i.e., those that led to statistically significant correlations with response times, see Table 4), divided in three blocks, were entered in the following order: Block 1, formed by variables assessing the personality component (Openness to Experience and Agreeableness of the BFO, and State Anxiety); Block 2, formed by measures of WM and inhibitory control (recalled words and intrusions of the Listening Span); and Block 3, formed by a variable testing crystallized verbal intelligence (Vocabulary) (see Table 5).

**Table 4:** Pearson two-tailed bivariate correlations for the variables with statistically significant correlations with the measures of idiom processing.

	**1**	**2**	**3**	**4**	**5**	**6**	**7**	**8**	**9**	**10**	**11**	**12**	**13**	**14**	**15**	**16**
1. LS_Recalled	1	−0.218	0.163	−0.136	−0.069	−0.098	−0.056	0.072	−**0.288**	−**0.343**	−**0.359**	−**0.295**	−**0.328**	−**0.337**	−**0.335**	−**0.328**
		0.083	0.199	0.282	0.592	0.45	0.668	0.576	**0.021**	**0.005**	**0.004**	**0.018**	**0.008**	**0.006**	**0.007**	**0.008**
		64	64	64	62	62	62	62	**64**	**64**	**64**	**64**	**64**	**64**	**64**	**64**
2. LS_Intrusions		1	−0.066	0.069	0.006	0.**259**	−0.019	**0.279**	**0.280**	0.182	0.133	0.223	0.234	0.185	0.206	0.208
			0.607	0.585	0.965	**0.042**	0.883	**0.028**	**0.025**	0.151	0.293	0.076	0.063	0.142	0.102	0.098
			64	64	62	**62**	62	**62**	**64**	64	64	64	64	64	64	64
3. Vocabulary			1	−0.173	0.122	0.195	0.192	0.139	−**0.311**	−0.226	−0.234	−**0.285**	−**0.272**	−**0.269**	−**0.276**	−**0.263**
				0.171	0.346	0.128	0.135	0.281	**0.012**	0.073	0.062	**0.022**	**0.029**	**0.031**	**0.027**	**0.036**
				64	62	62	62	62	**64**	64	64	**64**	**64**	**64**	**64**	**64**
4. STAI_State				1	0.196	0.222	0.096	0.219	−0.228	−0.218	−**0.294**	−**0.254**	−0.230	−**0.283**	−**0.271**	−**0.243**
					0.127	0.083	0.458	0.087	0.069	0.083	**0.019**	**0.043**	0.068	**0.024**	**0.030**	**0.053**
					62	62	62	62	64	64	**64**	**64**	64	**64**	**64**	**64**
5. STAI_Trait					1	**0.323**	0.119	**0.436**	−0.222	−0.219	−0.191	−**0.255**	−0.227	−0.232	−0.211	−0.244
						**0.012**	0.366	**0.0001**	0.082	0.087	0.136	**0.045**	0.076	0.070	0.10	0.056
						**60**	60	**60**	62	62	62	**62**	62	62	62	62
6. Extraversion						1	0.244	**0.546**	−0.241	−0.156	−0.177	−**0.272**	−0.200	−0.233	−0.212	−221
							0.056	**0.0001**	0.059	0.226	0.168	**0.032**	0.118	0.068	0.098	0.085
							62	**62**	62	62	62	**62**	62	62	62	62
7. Agreeableness							1	0.**364**	−0.212	0.145	−**0.276**	−**0.274**	−0.180	−**0.284**	−**0.253**	−0.216
								**0.004**	0.098	0.262	**0.030**	**0.031**	0.161	**0.025**	**0.047**	0.092
								**62**	62	62	**62**	**62**	62	**62**	**62**	62
8. Openness								1	−**0.268**	−0.175	−**0.323**	−**0.255**	−0.224	−**0.298**	−**0.306**	−0.221
									**0.035**	0.173	**0.010**	**0.046**	0.080	**0.019**	**0.016**	0.084
									**62**	62	**62**	**62**	62	**62**	**62**	62
9. P_Idiom.									1	**0.885**	**0.894**	**0.892**	**0.965**	**0.923**	**0.969**	**0.913**
										**0.0001**	**0.0001**	**0.0001**	**0.0001**	**0.0001**	**0.0001**	**0.0001**
										**64**	**64**	**64**	**64**	**64**	**64**	**64**
10. P_Control										1	**0.892**	**0.895**	**0.976**	**0.923**	**0.913**	**0.972**
											**0.0001**	**0.0001**	**0.0001**	**0.0001**	**0.0001**	**0.0001**
											**64**	**64**	**64**	**64**	**64**	**64**
11. UP_Idiom.											1	**0.872**	**0.920**	**0.966**	**0.978**	**0.906**
												**0.0001**	**0.0001**	**0.0001**	**0.0001**	**0.0001**
												**64**	**64**	**64**	**64**	**64**
12. UP_Control												1	**0.920**	**0.969**	**0.906**	**0.974**
													**0.0001**	**0.0001**	**0.0001**	**0.0001**
													**64**	**64**	**64**	**64**
13. P_Idioms													1	**0.951**	**0.966**	**0.973**
														**0.0001**	**0.0001**	**0.0001**
														**64**	**64**	**64**
14.UP_Idioms														1	**0.972**	**0.973**
															**0.001**	**0.0001**
															**64**	**64**
15.Idiom._Targets															1	**0.934**
																**0.0001**
																**64**
16.Control_Targets																1

*The first number in each cell represents the r value, the second the p-value and the third the number of observations entering in the correlation (in a few cases some observations were missing due to technical problems). Statistically significant correlations are in reported in bold*.

*LS_Recalled, number of correctly recalled words in the Listening Span; LS_Intrusions, number of intrusions in the Listening Span; Vocabulary, scores in the Vocabulary test; STAI_State, STAI raw scores in the State dimension; STAI_Trait, STAI raw scores in the Trait dimension; Extraversion, BFO raw scores of the Extraversion dimension; Agreeableness, BFO raw scores of the Agreeableness dimension; Openness, BFO raw scores of the Openness dimension; P_Idiom, Response times (RTs) to idiomatic targets of Predictable idioms; P_Control, RTs to control targets of Predictable idioms; UP_Idiom, RTs to idiomatic targets of Unpredictable idioms; UP_Control, RTs to control targets of Unpredictable idioms; P_Idioms, RTs to Predictable idioms; UP_Idioms, RTs to Unpredictable idioms; Idiom_targets, RTs to idiomatic targets; Control_Targets, RTs to control targets*.

**Table 5:** Descriptive statistics for the individual difference measures entered in the stepwise regression analysis.

**Variable**	**Observed range**	**Mean**	**Standard deviation**
**BLOCK 1**			
Openness to experience	14–35	24.64	5.04
Agreeableness	10–40	21.82	6.52
State anxiety	21–63	34.0	7.77
**BLOCK 2**			
LS: correctly recalled words	8–30	22.09	4.81
LS: Intrusion errors	0–6	1.98	1.62
**BLOCK 3**			
Vocabulary	28–58	42.03	7.48

Given the large number of tests, the alpha inflation was controlled by setting the False Discovery Rate to 0.05. The FDR was estimated through the procedure described in Storey and Tibshirani (2003). The bootstrap procedure was used to estimate the π0 parameter (Storey et al., 2004; for a general view on the bootstrap procedure see for instance Efron and Tibshirani, 1993; Di Nocera and Ferlazzo, 2000). In our analyses, the 0.05 levels of significance corresponded to an FDR < 0.05.

## Results

### Cross-modal lexical decision times experiment

**Figure 1:**
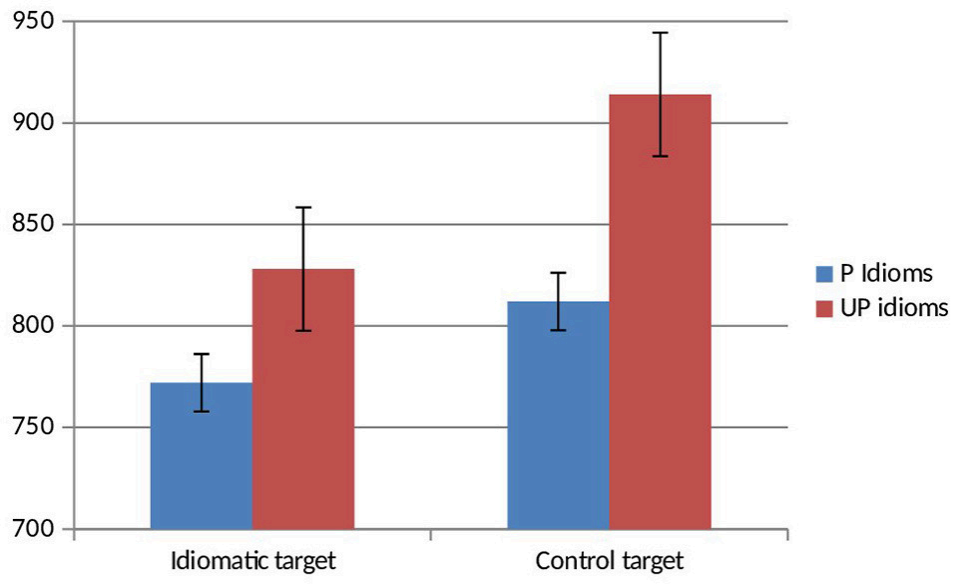
Means of the correct responses for idiomatic and control targets of predictable (P) and unpredictable idioms (UP idioms). Bars refer to standard error.

All participants met the threshold in the recognition test (mean correct recognition = 79%). In order to reduce variability, data points ±2 *SD*s from the mean response time of each participant were excluded from the analyses (1.3%). Errors represented only the 1.07% (*SD* = 0.58) of the total responses, therefore they were not statistically analyzed (see Figure 1 for the mean lexical decision times for idiomatic and control targets of predictable and unpredictable idioms).

The Target factor was significant [*F*_1_(1, 63) = 42.844, MSE 402590.25, η_*p*_^2^ = 0.405, *p* = 0.000; *F*_2_(1, 26) = 8.582, MSE 107976.446, η_*p*_^2^ = 0.248, *p* = 0.007; MinF′ (1, 37) = 7.336, *p* = 0.01] with faster lexical decision times for idiomatic than for control targets (95% CI: 65± ms). Unfortunately, both the Idiom type factor and the Idiom Type X Target interaction were significant in the by-participant but not in the by-item analysis [*F*_1_(1, 63) = 35.918, MSE 250357.141, η_*p*_^2^ = 0.363, *p* = 0.0001; *F*_2_(1, 26) = 2.212, MSE 20176.782, η_*p*_^2^ = 0.078, *p* = 0.149; MinF′ (1, 29) = 2.084, *p* = 0.16; *F*_1_(1, 63) = 3.877, MSE 32942.25, η_*p*_^2^ = 0.058, *p* = 0.053; *F*_2_(1, 26) = 0.191, MSE 2405.161, η_*p*_^2^ = 0.007, *p* = 0.666; MinF′ (1, 29) = 0.182, *p* = 0.673, respectively]. The relatively low number of stimuli per condition is presumably responsible for the lack of by-item significant effects in both cases.

Bonferroni-corrected *t*-tests showed that control targets were responded to significantly slower than idiomatic targets in both predictable and unpredictable idioms [*t*_(63)_ = 3.566, SEM 15.88, *d* = 0.50, *p* = 0.001; by an average of 57 ms; 95% CI: ±31.5 ms; *t*_(63)_ = 5.822, SEM 17.52, *d* = 0.83, *p* = 0.0001; by an average of 102 ms; 95% CI: ±35 ms, respectively]. Idiomatic and control targets were both responded to faster in predictable than in unpredictable idioms [*t*_(63)_ = 2.64, SEM 15.1, *d* = 0.36, *p* = 0.01; by an average of 40 ms; 95% CI: ±30 ms; *t*_(63)_ = 5.334, SEM 16, *d* = 0.69, *p* = 0.0001, by an average of 85 ms; 95% CI: ±32 ms, respectively].

In sum, we found contextually-driven activation of the idiomatic meaning for both predictable and unpredictable idioms in line with our prediction and with previous studies (e.g., Cacciari et al., 2007).

Following the suggestion of one the reviewers, we also used linear mixed models with random intercepts to investigate the effects of the Idiom type and Target factors, and their interaction. Idiom type, Target, and Idiom type by Target interaction were included as fixed effects in the model, whereas Item and Subject were included as random effects in the model. Before running the analyses, the small number of missing RTs were estimated through multiple imputations (R package “mice,” Buuren and Groothuis-Oudshoorn, 2011). Results replicated those showed above. Namely, removing the Target factor from the full model yielded a significant effect [$\chi_{\left(2\right)}^{2}$ = 42.89, *p* < 0.001], whereas removing the Idiom factor or the Idiom by Target interaction did not yield any significant effect [$\chi_{\left(2\right)}^{2}$ = 4.32, *p* = 0.12, and $\chi_{\left(1\right)}^{2}$ = 1.69, *p* = 0.19, respectively]. As the study was originally designed to be analyzed using the conventional by-participant and by-item ANOVA, we prefer to retain and discuss those results.

### Correlational analyses

All the variables included in the correlational analyses were normally distributed (Kolmogorov–Smirnov test > 0.10 in all cases). We only report the variables that led to statistically significant correlations with the measures of idiom processing (Table 4). For sake of clarity, we present each component separately.

#### WM and inhibitory control

Significant correlations only concerned the Listening Span test. Specifically, the number of recalled words inversely correlates with the response times to idiomatic and control targets of predictable (*r* = −0.288, *p* = 0.021, *r* = −0.343, *p* = 0.005, respectively) and unpredictable idioms (*r* = −0.359, *p* = 0.004; *r* = −0.295, *p* = 0.018, respectively). In sum, the faster participants were in correctly responding to idioms, the higher was their verbal WM. The number of intrusions is positively correlated with the response times to idiomatic targets of predictable idioms (*r* = 0.280, *p* = 0.025) in that the faster participants were in responding to idiomatic targets, the lesser intrusion errors they made.

#### Vocabulary

Vocabulary scores inversely correlate with the response times to idiomatic targets of predictable idioms (−0.311, *p* = 0.012), to predictable and unpredictable idioms (*r* = −0.272, *p* = 0.029; *r* = −0.269, *p* = 0.031, respectively), regardless of target type, and with the response times to idiomatic and control targets, regardless of idiom type (*r* = −0.276, *p* = 0.027; *r* = −0.263, *p* = 0.036, respectively). In sum, the better the participants performance was in the Vocabulary test, the faster participants were in responding to idioms.

#### Personality

State Anxiety scores inversely correlate with the response times to both idiomatic and control targets of unpredictable idioms (*r* = −0.294, *p* = 0.019; *r* = −0.254, *p* = 0.043, respectively), with the response times to unpredictable idioms, regardless of target type (*r* = −0.283, *p* = 0.024), and with the response times to idiomatic and control targets, regardless of idiom type (*r* = −0.271, *p* = 0.030; *r* = −0.243, *p* = 0.053, respectively). In sum, higher levels of temporary anxiety due to a specific event (presumably the experiment) were associated to faster response times to these targets. Trait Anxiety scores inversely correlate only with the response times to the control targets of unpredictable idioms (*r* = −0.255, *p* = 0.045). Extraversion scores of the BFO inversely correlate with the response times to control targets of unpredictable idioms (*r* = −0.272, *p* = 0.032). Both Agreeableness and Openness to Experience scores inversely correlate with the response times to idiomatic and control targets of unpredictable idioms (Agreeableness: *r* = −0.276, *p* = 0.03; *r* = −0.274, *p* = 0.031, respectively; Openness to Experience: *r* = −0.323, *p* = 0.01; *r* = −0.255, *p* = 0.046, respectively), with the response times to unpredictable idioms, no matter the target type (Agreeableness: *r* = −0.284, *p* = 0.025; Openness to Experience: *r* = −0.298, *p* = 0.019), and with the response times to idiomatic targets, no matter the idiom type (Agreeableness: *r* = −0.253, *p* = 0.047; Openness to Experience: *r* = −0.306, *p* = 0.016, respectively). Openness to Experience also inversely correlates with the response times to idiomatic targets of predictable idioms (*r* = −0.268, *p* = 0.035). In sum, the higher were the scores of Agreeableness and Openness to Experience, the faster were the response times to unpredictable idioms, and in general to control targets; the higher the scores of Openness to Experience, the faster the responses to idiomatic targets of predictable idioms, and lastly the higher the scores of Extraversion, the faster the responses to control targets of unpredictable idioms.

### Stepwise regression analyses

In the hierarchical regression analyses, significant effects were observed for the response times to idiomatic targets of predictable idioms. Block 2 (also including WM and Inhibitory control measures) accounted for 29.1% of the variance [*F*_(5, 61)_ = 4.603, *p* = 0.001, *R*^2^ = 0.291] with significant contributions of the constant term [β = 1537.309 (standard error = 212.10), *t*_(61)_ = 7.045, *p* < 0.001], and of the correctly recalled words and intrusions of the Listening Span [β = −0.246, *t*_(61)_ = −2.085, *p* = 0.042; β = 0.313, *t*_(61)_ = 2.564, *p* = 0.012, respectively]. Two further predictors had close-to-significance values: Openness to Experience and State anxiety [β = −0.250, *t*_(61)_ = 1.898, *p* = 0.063; β = −0.224, *t*_(61)_ = 1.912, *p* = 0.061, respectively]. Block 3 [also including Vocabulary, constant β = 1830.002, (standard error = 238.37)] accounted for an additional 6.6% of variance [*F*_(6, 61)_ = 5.987, *p* < 0.001, *R*^2^ = 0.357; β = −0.273, *t*_(61)_ = −2.369, *p* = 0.021]. The tolerance values (>7 for all the variables) and the variance inflation factor values (<1.5 for all the variables) did not suggest any multicollinearity in the analysis.

Significant effects of a single predictor (Vocabulary) were found in the response times to control targets of predictable idioms, where Vocabulary accounted for 10.1% of the variance [*R*^2^ = 0.101; β = 0.332, *t*_(61)_ = 2.441, *p* = 0.018], though the complete model was not significant [*F*_(6, 55)_ = 1.030, *p* = 0.416], and to idiomatic targets of unpredictable idioms, where Vocabulary accounted for 8.1% of the variance [*R*^2^ = 0.081; β = 0.277, *t*_(61)_ = 2.014, *p* = 0.049], though again the complete model was not significant [*F*_(6, 55)_ = 0.803, *p* = 0.572]. None of the Blocks or single predictors produced significant *R*^2^ changes in the response times to the other experimental conditions.

## General discussion

In line with prior evidence and with our predictions, when ambiguous idioms were embedded in idiom-biasing contexts the idiomatic meaning was equally available for predictable and unpredictable idioms. Clearly, the joint effects of the contextual bias and familiarity facilitated semantic processing making the idiomatic meaning available at the end of the idiom strings regardless of predictability.

The correlation and regression analyses showed important contributions of WM, inhibitory control and crystallized verbal intelligence, as well as marginally significant contributions of State Anxiety and Openness to Experience. These results represent an interesting, although still exploratory, addition to the literature and extend to idioms previous evidence about the components associated with metaphor and sarcasm comprehension (Blasko, 1999; Kazmerski et al., 2003; Chiappe and Chiappe, 2007; Carriedo et al., 2015; Olkoniemi et al., 2016; Kidd et al., 2018). Specifically, our regression analyses showed that WM and Inhibitory control (as estimated respectively by recalled words and intrusion errors of the Listening Span) accounted for a significant part of variance (29.1%) in the response times to idiomatic targets of predictable idioms. The Listening Span task (as other types of WM span tasks) requires the active maintenance of information in memory in face of concurrent processing and interference (Conway et al., 2003), and is considered to provide not only a measure of WM but also of language processing skills driven by linguistic experience (Farmer et al., 2017). This is particularly important to idioms since, as we mentioned, processing idioms may be more engaging for the cognitive system than it may appear given the rapidity with which familiar idioms are generally understood. In fact, it has been extensively shown that momentary activation of at least part of the literal meaning of the idiom composing words and retrieval, after idiom identification, of the idiom meaning from semantic memory are both necessary to idiom comprehension (Cacciari et al., 2007; Fanari et al., 2010; Vespignani et al., 2010; Holsinger and Kaiser, 2013; Canal et al., 2017). How many of the words composing the idiom string are literally processed depends on factors such as idiom predictability, familiarity and contextual information. It has been observed in fact that reading some of the words composing highly predictable familiar idioms may suffice to actively pre-activate the word(s) that are more likely to appear (Molinaro and Carreiras, 2010; Vespignani et al., 2010). At the same time, when the activated literal meanings are irrelevant to the idiomatic interpretation, these are actively suppressed (for a discussion on this point, see Rommers et al., 2013; Cacciari and Corradini, 2015). Fanari et al. (2010) showed that when short unpredictable idioms are embedded in idiom-neutral contexts, their figurative meaning was available only after the string offset. In this case, the literal meaning of the idiom composing words was fully activated. But when the same idioms were embedded in idiom-biasing contexts, idiom recognition was anticipated and the idiomatic meaning was already available at the string offset. Regression analyses revealed that the predicting role of inhibitory control was particularly evident only in the comprehension of predictable idioms. Admittedly, we do not have an explanation of the reasons why we did not find similar effects also on unpredictable idioms. One possibility is that effects of inhibitory control manifest themselves particularly with faster response times. Another, not necessarily alternative possibility, is that individuals scoring higher in WM and inhibitory control were more likely to possess a more robust knowledge about word co-occurrence which further speeded up the response times to predictable idioms. Future studies specifically devoted to test these possibilities are needed.

Vocabulary scores contributed to the response times to predictable and unpredictable idioms. The Vocabulary subtest of WAIS-R provides an estimate of global verbal intelligence function (Lezak et al., 2004) and of vocabulary size, an important predictor of language acquisition and processing (Kidd et al., 2018). Many studies documented an association of vocabulary knowledge to efficient sentence comprehension (Hunt, 1977). For instance, Kazmerski et al. (2003) found that vocabulary knowledge and comprehension abilities correlated and predicted metaphor interpretation in association to verbal WM. Similarly, Chiappe and Chiappe (2007) found that vocabulary knowledge (together with inhibitory control, and print exposure) predicted the quality of generated metaphors (see also Beaty and Silvia, 2013). We documented the important contribution of crystallized verbal intelligence also on idiom comprehension. To the best of our knowledge, few studies on idiom comprehension reported similar evidence. For instance, in a study testing idiom comprehension in people with paranoid schizophrenia and matched controls, Pesciarelli et al. (2014) reported that high scores in the Vocabulary subtest of WAIS contributed to increase response accuracy.

Stepwise regression analysis suggests a role also of personality-related variables. As the marginally significant effect of State Anxiety suggests, a temporary state of anxiety, presumably related to the experimental setting, was associated with faster response times to idiomatic targets of predictable idioms (the cognitively easiest experimental condition). Presumably, this (unpleasant) temporary emotional arousal led participants to recruit more cognitive resources speeding up idiom processing (as documented for literal language by Svenson and Maule, 1993; Breznitz and Berman, 2003). Openness to Experience as well (although again in a marginally significant way) contributed to the response times to idiomatic targets of predictable idioms. Recently, Beaty and Silvia (2013) reported that Openness to Experience contributed to creative metaphor quality (see also Goetzman et al., 2007). Several studies have suggested that Openness to Experience (and also Extraversion, in some studies) has a positive association with creativity in different domains (for an overview, see Batey and Furnham, 2006). This is the first time, to the best of our knowledge, that an association of Openness to Experience with the comprehension of conventionalized figurative strings is reported.

Surprisingly enough, we did not observe specific contributions of Digit Span and Stroop tests, and of verbal fluency and fluid intelligence tests that were instead reported in prior studies on figurative language comprehension. However, these effects were reported for metaphor interpretation and production (e.g., Chiappe and Chiappe, 2007; Silvia and Beaty, 2012). Metaphors and idioms differ in important respects mostly related to processing workload: first, (familiar) idioms possess a ready-to-go meaning that has to be retrieved from semantic memory, and not fully composed as in metaphors. The less demanding workload on the processing system necessary to compute idioms, compared to metaphors, together with the specific characteristics of our idiom experiment, can have made the contribution of these tests less visible than in metaphor studies. Again, specific studies testing different forms of figurative language are required to test this possibility. Even more strikingly, we did not find any effects of general speed of processing, at variance with a previous idiom study (Cacciari et al., 2007). However this may reflect methodological differences between the two studies.

Finally, one might wonder whether we can derive alternative predictions on the role of individual differences in idiom comprehension from Lexical Look-Up models and from Non-Lexical models. These models were not designed to take individual differences into account. Notwithstanding, it seems fair to hypothesize that specific contributions of cognitive and personality components would be best accommodated by Non-Lexical Hybrid models of idiom processing since these assume that idioms are processed just like any other linguistic input, up to idiom identification after which the idiom meaning is retrieved from semantic memory, and the activated literal meanings decay. These models would at least predict a role for WM and inhibitory control in idiom comprehension since some literal computation precedes idiom retrieval, and in most cases the literal meanings of the words activated prior to idiom recognition must be actively suppressed. It is more difficult to detail which of the components examined in the present study would be associated to idiom comprehension according to Lexical Look-up models that posit that idioms are computationally similar to long words. In this view, in fact idiomatic meanings do not undergo any linguistic computation being directly retrieved from semantic memory as for any other words.

## Conclusions

Individual differences in WM capacity, inhibitory control, crystallized verbal intelligence, State Anxiety, and Openness to Experience indeed seem to be associated with the contextually-driven comprehension of an important part of multi-word expressions, namely idioms. This is the picture emerging from this exploratory study. Admittedly, the correlational approach of the study has important limitations. In fact, we cannot a priori exclude that some or all the significant correlations found in this study may be due to higher level factors whose specific nature is not yet clear.

Studies with a higher number of stimuli (and also matched literal sentences) and participants testing specific hypothesis about each component of the cognitive and personality architecture underlying language processing are required before we can ultimately decide whether the complex architecture subserving idiom processing totally or partially coincides with that used to comprehend literal language, and what are the roles and weights of each component in idiom comprehension. One might also wonder whether these results are specific to idioms or they are generalizable to other kinds of literal and non-literal word strings. Again this is an empirical question that requires future studies to be properly answered.

Since this study used ambiguous idioms, the disambiguation of the idiom meaning required the presence and use of contextual information to decide which of the two meanings of the string was appropriate. Of course providing idiom-consistent information has facilitated idiomatic meaning activation speeding up response times (see Fanari et al., 2010). Future studies on the role of individual differences in idiom comprehension may adopt a different approach testing unambiguous idioms, presented in and out of context, with different behavioral paradigms (e.g., eye-tracking, sentence, or word-by-word reading times) that may provide a more detailed picture than the cross-modal lexical decision task that taps on whether or not the idiomatic meaning is available at the string offset.

Why it is important to study the role of individual differences in language acquisition and processing not relegating them to error variance (Kidd et al., 2018)? First, because the presence of individual differences obscured by group data may be one of the causes of inconsistent findings in the cognitive science literature (Hannon and Daneman, 2001) and of unresolved theoretical controversies in psycholinguistics (Kidd et al., 2018). Second, studying the role of individual variability may shed further light on the neural and cognitive architecture underlying language (and in general any mental processes) and on how it interacts with language experience. A related question is why idioms are relevant to these aims. The response is that they indeed are relevant not simply because they are pervasive in everyday communication, but rather because the study of individual differences in idiom comprehension can provide a better understating of the role of distributional information in language processing. Interestingly, the relevance of the notion of predictability in contemporary models of language processing (for an overview, see Kutas and Federmeier, 2000) has led to an increasing interest in the mechanisms underlying idiom comprehension (Vespignani et al., 2010; Cacciari, 2014). Idiomatic expressions, together with other types of multi-word literal and non-literal expressions, represent an interesting test case of how the brain and the mind handle the frequency with which we are exposed to linguistic input in the environment (statistical learning). In fact, the brain is sensitive to distributional information in the input as reflected, for language, by the frequency of co-occurrence of words in compositional (e.g., *black and white*) and non-compositional multi-word strings (e.g., *kick the bucket*) (e.g., Arnon and Snider, 2010; Molinaro and Carreiras, 2010; Tremblay and Baayen, 2010; Vespignani et al., 2010; Tremblay et al., 2011; Siyanova-Chanturia, 2013; Cacciari and Corradini, 2015).

## Author contributions

CC initially conceived the idea for the study which was then further developed and finalized by CC and PC. The stimulus materials were prepared by PC. Data collection was made by PC. Analyses were run by CC, FF, and PC. The majority of this paper was written by CC.

### Conflict of interest statement

The authors declare that the research was conducted in the absence of any commercial or financial relationships that could be construed as a potential conflict of interest.

## Footnotes

^1^Prefrontal and frontal regions are involved in the retrieval of the idiomatic meaning from semantic memory, in response monitoring, and in the inhibition of an alternative interpretation. Selection and monitoring of internally generated responses are likely to be performed by the central executive whose neural correlates predominantly are in the prefrontal lobe (Oliveri et al., 2004; Fogliata et al., 2007; Rizzo et al., 2007; Romero Lauro et al., 2008; Cacciari and Papagno, 2012).

## Appendix

PREDICTABLE IDIOMS

1. *Ficcare il naso*

La cameriera ha visto l'agenda aperta sul tavolo e ci ha ficcato il naso.

CURIOSITA' / DIFFICOLTA'

2. *Aprire gli occhi*

Anna si era innamorata dell'uomo sbagliato, ma ora finalmente ha aperto gli occhi.

CONSAPEVOLEZZA / QUALIFICAZIONE

3. *Prendere al volo*

Il cliente ha ascoltato l'offerta del venditore e ha deciso di prenderla al volo.

OCCASIONE / DESTINO

4. *Pugnalare alle spalle*

Il rapinatore disse alla polizia il nome del complice e così lo pugnalò alle spalle.

TRADIMENTO / RENDIMENTO

5. *Rimanere sullo stomaco*

A Giorgio la decisione di sospendere gli aumenti di stipendio è rimasta sullo stomaco.

RABBIA / STAMPA

6. *Sporcarsi le mani*

Quando il noto deputato ha accettato la grossa tangente si è davvero sporcato le mani.

DISONESTA' / ANDAMENTO

7. *Strapparsi i capelli*

Quando Luisa si è accorta che aveva perso l'antico anello si è strappata i capelli.

DISPERAZIONE / RELAZIONE

8. *Stringere i denti*

Per arrivare alla fine della lunga maratona il podista ha dovuto stringere i denti.

SFORZO / ROVESCIO

9. *Tapparsi le orecchie*

Il medico diceva sempre a Luca di smettere di fumare ma lui si tappava le orecchie.

RIFIUTO / RISATA

10. *Rimboccarsi le maniche*

Per terminare in tempo la relazione per il direttore, l'impiegata si è rimboccata le maniche.

IMPEGNO / PASSAGGIO

11. *Sfuggire di mano*

Il poliziotto doveva mantenere l'ordine nel corteo, ma la situazione gli è sfuggita di mano.

CONTROLLO / INDIRIZZO

12. *Vuotare il sacco*

Dopo avere a lungo negato le accuse, alla fine il ragazzo ha vuotato il sacco.

CONFESSIONE / SEZIONE

13. *Brancolare nel buio*

La polizia stava indagando sull'omicidio da mesi, ma continuava a brancolare nel buio.

INCERTEZZA/ COLAZIONE

14. *Voltare le spalle*

Il padre sapeva che il figlio era in crisi, ma ugualmente gli ha voltato le spalle.

INDIFFERENZA / RICOVERO UNPREDICTABLE IDIOMS

15. *Giocare in casa*

Lucia ha fatto il concorso nella sua Università perché sapeva di giocare in casa.

VANTAGGIO / SVOLTA

16. *Perdere il filo*

Sara stava raccontano una favola alla nipotina ma si accorse di aver perso il filo.

DISTRAZIONE / NAZIONE

17. *Rompere il ghiaccio*

Fabio voleva uscire con la nuova studentessa francese e ha cercato di rompere il ghiaccio.

IMBARAZZO / REGIME

18. *Scendere in campo*

Dopo i gravi episodi di razzismo molte associazioni per i diritti umani sono scese in campo.

AZIONE / COPPIA

19. *Tagliare la corda*

Il rapinatore non fece in tempo a scassinare la cassaforte e dovette tagliare la corda.

FUGA / LEZIONE

20. *Prendere di petto*

Matteo aveva un serio problema di salute e senza aspettare l'ha preso di petto.

DECISIONE / RUMORE

21. *Dare una strigliata*

Pietro è stato di nuovo bocciato all'esame di inglese e sua madre gli ha dato una strigliata.

RIMPROVERO / RISERVATEZZA

22. *Essere a cavallo*

Quando la studentessa di ingegneria elettronica terminò tutti gli esami capì di essere a cavallo. RIUSCITA / GIOVINEZZA

23. *Toccare il fondo*

Andrea ha perso tutti i soldi al gioco d'azzardo ed ora ha veramente toccato il fondo.

ROVINA / RIFUGIO

24. *Alzare il gomito*

Ieri sera gli amici sono andati in osteria e qualcuno di loro ha alzato il gomito.

UBRIACHEZZA / INTROSPEZIONE

25. *Mettersi in mezzo*

Dato che i parenti stavano litigando per la divisione dell'eredità Carla si è messa in mezzo.

INTROMISSIONE / INEFFICIENZA

26. *Fare i conti*

La mamma era molto arrabbiata col figlio e gli ha detto che quella sera avrebbero fatto i conti.

SGRIDATA / PREPARAZIONE

27. *Togliersi dai piedi*

Mara non sopportava più la suocera troppo invadente e voleva assolutamente togliersela dai piedi.

FASTIDIO / CONTEGNO

28. *Chiudere un occhio*

La maestra si era accorta che Giulio aveva copiato dal compagno, ma ha chiuso un occhio.

TOLLERANZA / RILASCIO
